# Analysis of proteome response to the mobile phone radiation in two types of human primary endothelial cells

**DOI:** 10.1186/1477-5956-8-52

**Published:** 2010-10-18

**Authors:** Reetta Nylund, Niels Kuster, Dariusz Leszczynski

**Affiliations:** 1 STUK - Radiation and Nuclear Safety Authority, Helsinki, Finland; 2 IT'IS Foundation, Swiss Federal Institute of Technology, Zurich, Switzerland

## Abstract

**Background:**

Use of mobile phones has widely increased over the past decade. However, in spite of the extensive research, the question of potential health effects of the mobile phone radiation remains unanswered. We have earlier proposed, and applied, proteomics as a tool to study biological effects of the mobile phone radiation, using as a model human endothelial cell line EA.hy926. Exposure of EA.hy926 cells to 900 MHz GSM radiation has caused statistically significant changes in expression of numerous proteins. However, exposure of EA.hy926 cells to 1800 MHz GSM signal had only very small effect on cell proteome, as compared with 900 MHz GSM exposure. In the present study, using as model human primary endothelial cells, we have examined whether exposure to 1800 MHz GSM mobile phone radiation can affect cell proteome.

**Results:**

Primary human umbilical vein endothelial cells and primary human brain microvascular endothelial cells were exposed for 1 hour to 1800 MHz GSM mobile phone radiation at an average specific absorption rate of 2.0 W/kg. The cells were harvested immediately after the exposure and the protein expression patterns of the sham-exposed and radiation-exposed cells were examined using two dimensional difference gel electrophoresis-based proteomics (2DE-DIGE). There were observed numerous differences between the proteomes of human umbilical vein endothelial cells and human brain microvascular endothelial cells (both sham-exposed). These differences are most likely representing physiological differences between endothelia in different vascular beds. However, the exposure of both types of primary endothelial cells to mobile phone radiation did not cause any statistically significant changes in protein expression.

**Conclusions:**

Exposure of primary human endothelial cells to the mobile phone radiation, 1800 MHz GSM signal for 1 hour at an average specific absorption rate of 2.0 W/kg, does not affect protein expression, when the proteomes were examined immediately after the end of the exposure and when the false discovery rate correction was applied to analysis. This observation agrees with our earlier study showing that the 1800 MHz GSM radiation exposure had only very limited effect on the proteome of human endothelial cell line EA.hy926, as compared with the effect of 900 MHz GSM radiation.

## Background

The use of mobile phones has widely increased over the past decade. In spite of the extensive research, the question of the possible health effects of the mobile phone radiation remains open. In 2001 we have proposed [1] and subsequently demonstrated [2] that proteomics could be used as a tool to find the protein targets that are affected by the mobile phone radiation. Based on the knowledge which proteins respond to the mobile phone radiation, new hypotheses about the possible biological effects might be put forward for testing. So far, the proteomics approach has been used only in a very few studies examining effects of the mobile phone radiation [2-10]. Therefore, based on this very limited material, it is not yet possible to draw any general conclusions about the effects of this radiation on cell proteome or on the physiological processes regulated by the affected proteins.

In our earlier studies we have determined that the 900 MHz GSM mobile phone radiation induces proteome changes in human endothelial cell line EA.hy926 [2-5]. Furthermore, it appears that cell response to this radiation might depend on the transcriptome and proteome expressed by the cells at the time of exposure [5,11]. Using two variants of the EA.hy926 cell line, we have observed that the variants responded differently, on transcriptome and proteome level, to the same 900 MHz GSM signal [5]. On the other hand, we have observed that exposure of EA.hy926 cells to 1800 MHz GSM radiation had very low, if at all, statistically significant effect on cell proteome [8]. Therefore, it is unclear whether 900 MHz and 1800 MHz GSM radiation differ in their ability to induce biological effects.

In the present study, using primary human endothelial cells derived from two different vascular beds, we have examined cell responses to 1800 MHz GSM signal of mobile phone radiation. The examined cells were primary human umbilical vein endothelial cells (HUVEC) and primary human brain microvasculature endothelial cells (HBMEC). Both of the primary endothelial cell types were exposed for 1 hour to the 1800 MHz GSM mobile phone radiation at an average specific absorption rate (SAR) of 2.0 W/kg and harvested, as in our earlier studies [2-5,8], immediately after the end of exposure. The protein expression patterns in both cell types were examined using two dimensional difference gel electrophoresis (2D DIGE) -based proteomics [12].

## Materials and methods

### Cell culture and conditions

**Figure 1:**
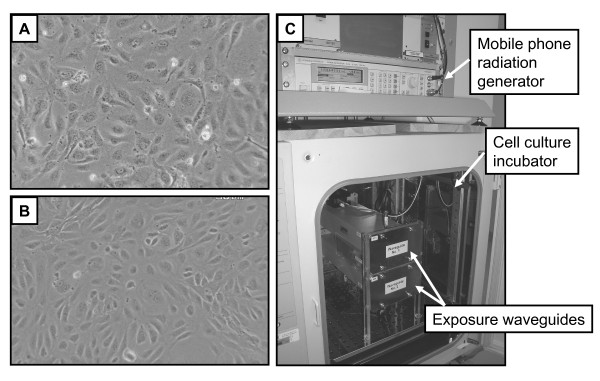
**Monolayers of HUVEC (A) and HBMEC (B)**. **Exposure set-up for 1800 MHz GSM mobile phone radiation (C).**

Primary human umbilical vein endothelial cells (HUVEC) were purchased form Lonza, Switzerland and cultivated according to manufacturer's instructions. The purchased HUVEC were a pool of cells from several donors. For mobile phone radiation experiments, cells were removed from culture flasks by brief trypsinization, washed in cell culture medium and seeded in the 35 mm-diameter "CellBIND" Petri dishes (Corning, USA). After overnight incubation the medium in the dishes was replaced with a fresh one and the monolayers of HUVEC (Figure 1A) were exposed to the mobile phone radiation in a special exposure chamber. The sham samples (unexposed control) were produced simultaneously in an identical sham exposure chamber (see below description of the system). Immediately after the end of exposure the cells were quickly washed with warm (37°C) PBS and harvested with warm versene solution. In total, 13 independent sham and exposed samples were generated from HUVEC in 13 different exposure experiments.

Primary human brain microvascular endothelial cells (HBMEC) were purchased from ScienCell Research Laboratories, USA and cultivated according to manufacturer's instructions. The purchased HBMEC were from a single donor and all cells used for the experiments were from the same batch. Before experiments cells were grown to confluency, detached with trypsin and seeded in the 35 mm-diameter "CellBIND" Petri dishes (Corning, USA) that were additionally coated with fibronectin (1.5%, overnight at 37°C) (Sigma, USA). Seventy-two hours after seeding the medium was replaced with a fresh one and the monolayers of HBMEC (Figure 1B) were exposed to the mobile phone radiation using the same exposure chamber as for HUVEC. Immediately after the end of exposure the cells were rinsed with warm PBS and harvested with trypsin. In total, 11 independent sham and exposed samples were generated from HBMEC in 11 different exposure experiments.

### Mobile phone radiation exposure

The sXc-1800 exposure system, developed and provided by the IT'IS Foundation (Zurich, Switzerland) was used for exposing cells to 1800 MHz GSM signal (Figure 1C). The detailed description of the system and the dosimetry of it have been presented elsewhere [13]. Briefly: The system consists of two identical exposure chambers mounted inside the same cell culture incubator (NuAire US Autoflow CO_2 _Water-Jacketed Incubator, NuAire, USA). One of the chambers acted as a sham control (no radiation) and the other as an experimental (with radiation). Sham exposure chamber and RF exposure chamber were randomly assigned by the computer program that controlled exposures. This computer program has generated during the experiment encrypted files with information in which of the two chambers was radiation and which acted as sham-control. These encrypted files were decoded after the experiment by chamber manufacturer, IT'IS, Zurich, Switzerland. This set-up permitted blinded execution of experiments. The exposure system is fully automated and enables controlled exposures of cells (H-polarization or at H-field maximum of the standing wave [14]) at freely programmable amplitude modulations. Identical environmental conditions existed in both chambers (sham and experimental) since they were both located in the same cell culture incubator and the inlets of the airflow through them are at the same location. At 10 seconds intervals the system has monitored the incident field strengths, the proper functioning of the ventilators, the outlet air temperatures and the state of all equipment. The Pt100 temperature sensors (accuracy ± 0.1 °C) have been calibrated prior to the installation and the recorded differences in temperature are well within the specified long-term stability of the calibration. The induced temperature load, due to radiation absorption, has been characterized as a function of SAR (t) for different signals and volumes of medium. This enables a reliable estimate of the maximum temperature rise as a function of the exposure [13]. SAR distribution within the cell culture dish was characterized with a full three-dimensional 83-D) electrothermal finite-difference time-domain (FDTD) analysis using the simulation platform SEMCAD (by SPEAG). Additionally, SAR intensity and distribution was verified with measurements using a 1-mm-diameter-field probe inserted into the culture medium of the cell culture dish [13]. The simulated mobile phone signal used in this study was 1800 MHz GSM Talk-signal. It is characterized by a random change between the discontinuous transmission mode (DTX) and non-DTX or GSM Basic phases. The distribution in time was exponential with a mean duration of 10.8 seconds for non-DTX ("talking") and 5.6 seconds for DTX ("listening"). The dominant modulation components of this signal are 2, 8, 217, 1733 Hz and higher harmonics [15].

The monolayers of primary human endothelial cells were placed to two 6-dish holders and placed inside the exposure chambers of the exposure set-up. In one chamber, randomly selected by the computer program, cells were exposed to an average SAR of 2.0 W/kg at 37 ± 0.3°C for 1 hour, while in the other chamber the cells were sham-exposed in the similar conditions but without mobile phone radiation exposure. The experiments were performed in the blinded manner and the code was broken at IT'IS after the analyses of the experiments were completed.

### Sample preparation & labeling for 2-dimensional electrophoresis

Cell pellets were lysed in 50 μl of a lysis buffer (8 M Urea, 1 M Thiourea, 4% Chaps, 30 mM Tris, 1 mM sodium orthovanadate, and 1 mM PMSF, pH 8.5) for 1 hour at the room temperature, followed by centrifugation (twice) for 15 min at 20000 g each. Protein concentrations were measured using Bradford method. The 75 μg of total protein from each sample was used for two-dimensional gel electrophoresis. The internal standard was prepared by pooling of the same amount of each sample into a one common internal standard sample.

The 75 μg of total protein from each sample was used for the analysis. Samples were labeled with DIGE Cy-fluorescent dyes (GE Healthcare, USA) and internal standard sample was labeled with Cy2 dye in all cases. Each experimental sample was labeled with either Cy3 or Cy5 dye. The coding of the sample labeling was according to the exposure chamber: cells placed in chamber #1 of the set-up were always labeled with Cy3 dye and cells placed in chamber #2 were always labeled with Cy5 dye. The labeling procedure was done according to the manufacturer's instructions. Briefly: 600 pmol of dye was added to the sample and labeling was performed for 30 min on ice. Afterwards the labeling was quenched with 10 mM lysine for 10 minutes on ice. Samples deriving from the same exposure (Cy3 and Cy5 labeled) were pooled together with Cy2 labeled internal standard and separated all together in a single 2DE gel.

### 2-dimensional electrophoresis

The isoelectric focusing was performed using an IPGphor3 apparatus (GE Healthcare) and 24 cm long IEF strips pH 4-7 (GE Healthcare). The samples were loaded using in-gel rehydration loading in a buffer containing 9 M Urea, 2% Chaps, 0.5% IPG buffer pH 4-7, and 65 mM DTT for 5 h. IEF was run with 50 μA/strip at 20°C using step-and-hold method as follows: 50 V 8 h; 100 V 1 h; 500 V 1 h; 1000 V 1 h; 2000 V 1 h; 5000 V 1 h, 10000 V until 95000 Vhrs were achieved. After the end of IEF run the strips were equilibrated for 15 min with 6 M urea, 30% glycerol, 50 mM Tris-HCl, 2% SDS, and 10 mg/mL DTT for 15 min and then for another 15 min in the same buffer, in which 25 mg/mL iodoacetamide (IAA) has replaced DTT. SDS-PAGE was run in 10% gel using Ettan DALTsix Electrophoresis system (GE Healthcare) using 1 mm low fluorescent glass plates with the constant settings of 10 mA/1W/gel for the first hour and then 12 mA/1.5W/gel overnight at 20°C. After the electrophoresis the gels were scanned between the glass plates with Typhoon Trio scanner (GE Healthcare) with the appropriate excitation and emission wavelengths for Cy2, Cy3, and Cy5 dyes. The PMT voltages were optimized in such manner that the maximum signal intensity was approximately on the same level for all dyes.

### Data acquiring and analysis

The images were acquired with Typhoon Trio scanner (GE Healthcare). The datasets containing images from Cy3, and Cy5 labeled samples and Cy2 labeled internal standard were cropped with ImageQuant tool-software (GE Healthcare). The datasets were cropped to contain the same pattern of proteins in all cases. The datasets were then imported to DeCyder 6.5 software (GE Healthcare), in which the batch processor was used to detect and to match the spots. The 10000 spots were assumed to be found in the spot detection, and the volume of 30000 was used as a cut-off filter. After a brief manual visual check of the matched spots the workspace was imported to DeCyder Extended Data Analysis module (EDA) for statistical analysis. For EDA analysis protein spots, which were found at least in 70% of gels, were included. The student t-test was used to find differentially expressed protein spots. False discovery rate correction (FDR) was applied when t-test was performed in EDA module. Also principal component analysis (PCA) was performed in EDA for the spot maps. The lists of the statistically significantly affected spots were imported back to DeCyder Biological Variation Analysis module (BVA) in which the results were filtered on the basis of the average ratio between the samples.

## Results

Two different types of primary human endothelial cells were used in this study, the HUVEC and the HBMEC. The protein expression patterns of these cells were examined using 2DE-based proteomics with DIGE-technique. In total, 13 separate replicates of sham and exposed proteomes were generated from HUVEC and 11 separate replicates of sham and exposed proteomes from HBMEC. The same internal standard was used for all samples allowing better technical quality and less variance between the gels. All gel images were analyzed together in DeCyder 6.5. In total, 2863 protein spots were detected in the master gel. Protein spots which were detected in 70% of spot maps were included in the EDA analysis (total of 1746 spots).

**Figure 2:**
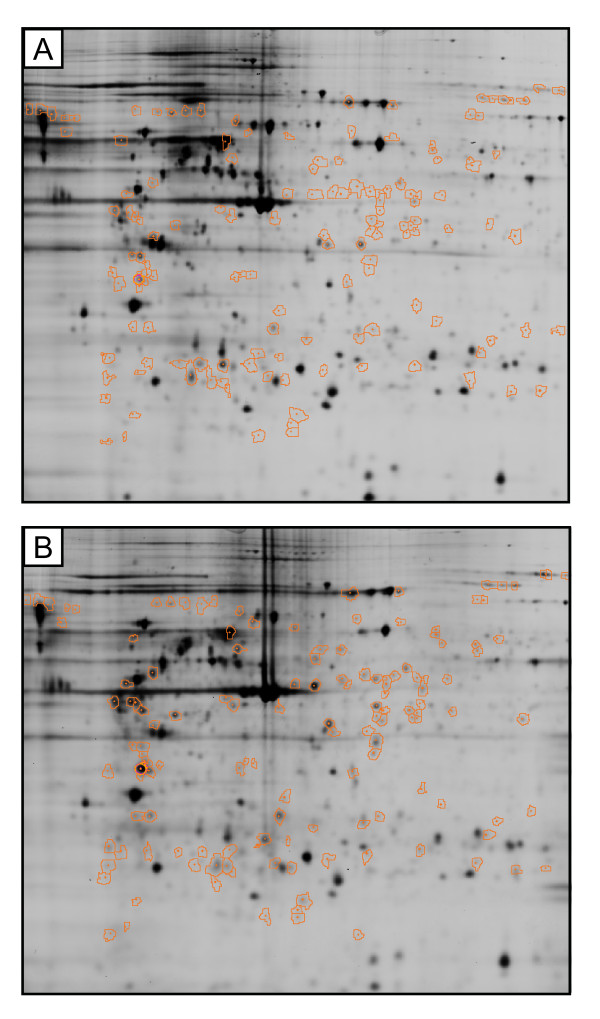
**2D-gels of HUVEC (A) and HBMEC (B) with marked 145 spots (orange colored rings) that are differing between the cell types**.

The proteome analysis has shown differences in 2D protein expression pattern between HUVEC and HBMEC. In total, 368 spots were found to differ between both cell types using an independent t-test with p ≤ 0.0001 and with false discovery rate correction. Out of these 368 protein spots, the 145 spots were found to be differentially expressed between the cell types by more than 2-folds up or down (Figure 2).

**Figure 3:**
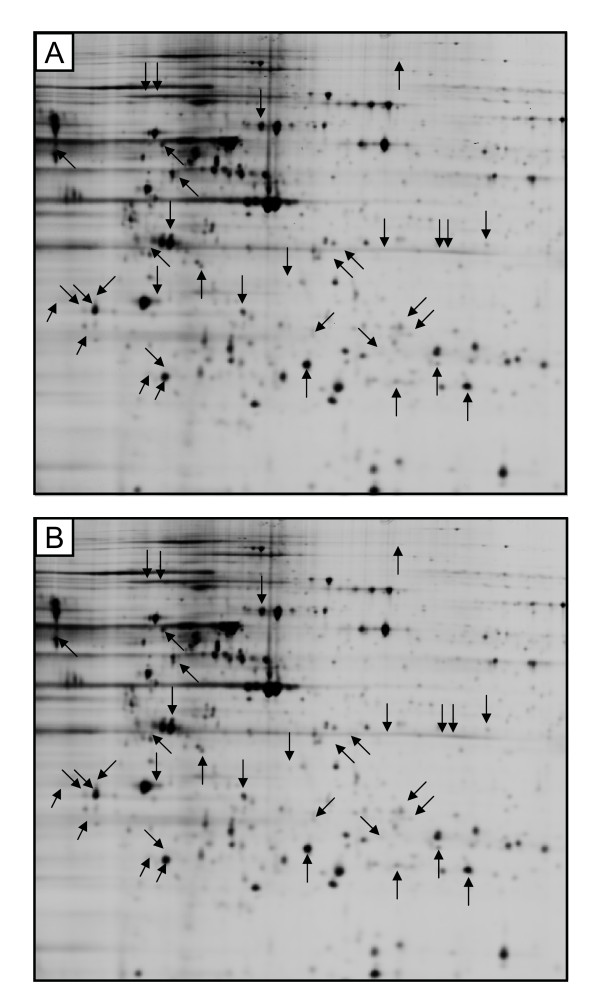
**2D-gels of HUVEC cells that were either sham (A) or RF-EMF exposed (B)**. Arrows point to 35 affected spots; before FDR correction.

**Figure 4:**
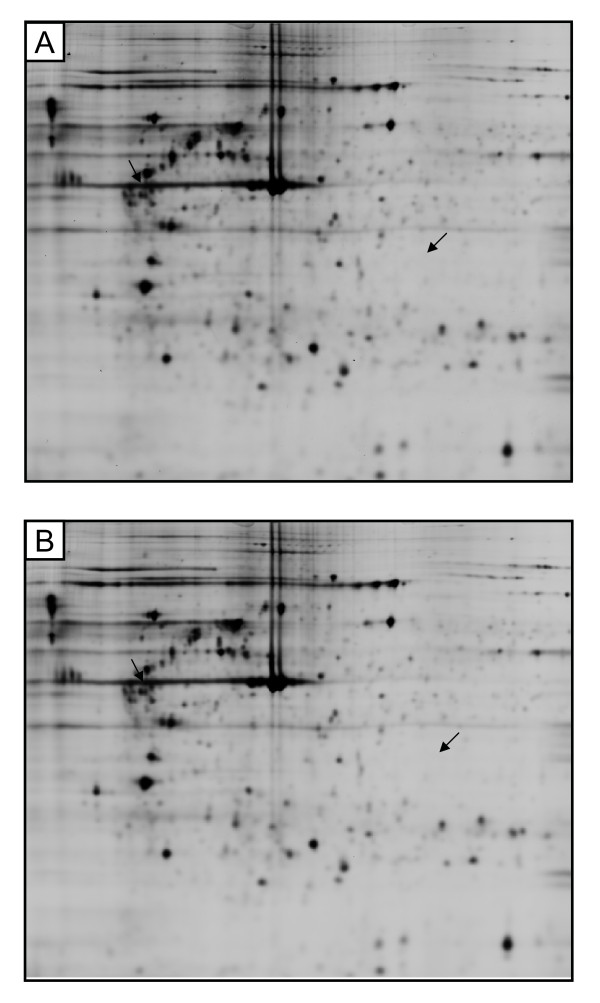
**2D-gels of HBMEC cells that were either sham (A) or RF-EMF exposed (B)**. Arrows point to two affected spots; before FDR correction.

Based on our previous study [4], it was expected that the different physiological properties of HUVEC and HBMEC, may lead to the induction of different protein expression profiles following the exposure to mobile phone radiation. In both cell types the differences in the protein expression in the response to the mobile phone radiation were analyzed for the 1746 spots included in EDA analysis, using independent t-test. In HUVEC proteome there were found 35 statistically significantly affected protein spots (p ≤ 0.05; t-test) (Figure 3). The maximum average ratio, between sham and exposed samples, was for these protein spots = 1.33. In HBMEC proteome there were found 2 statistically significantly affected protein spots (p ≤ 0.05; t-test) and the average ratios of -1.16 and +1.1 were observed between sham and exposed samples (Figure 4).

However, when the false discovery rate correction (FDR) was performed, all the statistically significantly affected spots were recognized as false positives. This outcome of FDR analysis might be explained by the low average ratio between exposed and sham samples (difference considered as a noise) or because some of the protein spots have appeared in manual visual examination as technical artefacts (e.g. dust particles). Indeed, all spots, found to be differentially expressed before FDR analysis, were also manually checked and the average ratios between exposed and sham samples were shown to be very close to 1.0 and the highest average ratio peaks were recognized as dust particles due to extremely sharp peak geometry.

**Figure 5:**
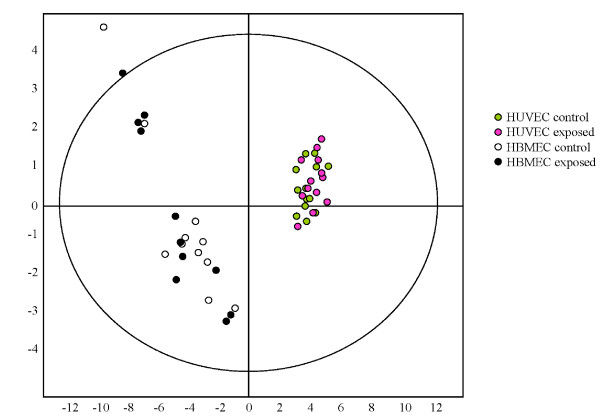
**Principal component analysis (PCA) of the protein spot maps shows the majority of differences to be found between the cell types, but not between the exposure conditions**.

Also a principal component analysis (PCA) of the protein spot maps was performed in EDA. The comparison of the protein spot maps showed that the first principal component in the analysis was clearly set as cell type, and not the exposure condition (Figure 5). Additionally, analysis has shown that in HBMEC there is a great dispersion in protein maps between individual exposures (replicates). Thus, PCA also demonstrates that the differences were found only between the cell types (analysis of which was not the aim of the study) but not between the exposure conditions (aim of the study).

## Discussion and Conclusions

Based on our earlier study [5] it was hypothesized that the endothelial cells, derived from the different vascular beds and having differing proteomes, would respond differently to mobile phone radiation exposure. At the same time, the study was to determine whether the observed earlier [8] very limited effect of 1800 MHz GSM radiation on the proteome of EA.hy926 human endothelial cell line will be reproduced using primary human endothelial cells.

As the proteome analysis has demonstrated, there are numerous differences in protein expression between proteomes of HUVEC and HBMEC. These differences are most likely reflecting the differences in physiological functions performed by endothelial cells in different vascular beds. The differences between proteomes of primary cells remained in cultures for several *in vitro *passages, indicating that they might be of significance for the specific cell functions and not just transient alterations of the dynamic proteome.

The exposure of HUVEC and HBMEC to 1800 MHz GSM mobile phone radiation did not cause any statistically significant changes in proteomes of either of cell types. This result differs from our earlier published studies where human endothelial cell lines (EA.hy926 and EA.hy926v1) were exposed to 900 MHz GSM mobile phone signal and statistically significant changes in proteome were detected [2-5]. However, this result agrees with our recent study [8] showing that the 1800 MHz GSM radiation has very small effect, if at all, on the proteome of EA.hy926 cell line, as compared with the 900 MHz GSM radiation.

The discrepancy between the responses of cells, to 1800 MHz GSM signal and the 900 MHz GSM signal, observed in our previous and in the current study, might be likely caused by: (i) different exposure frequencies (900 MHz vs. 1800 MHz), (ii) differences in SAR distribution in cell culture dishes in the used exposure set-ups, (iii) differences in used cell types (primary cells vs. cell line), and (iv) differences in the 2DE proteomics methodology (silver stain vs. DIGE).

In the 900 MHz GSM set-up there is a more non-uniform SAR distribution [3] than in the 1800 MHz set-up and therefore, cells in the certain areas of the culture dish are exposed to higher SAR (over 5.0 W/kg) when the average SAR for the whole cell culture dish is 2.4 W/kg [3]. The 1800 MHz GSM set-up has more uniform SAR distribution and the vast majority of cells, throughout the cell culture dish, were exposed to the same level of radiation SAR = 2.0 W/kg. Thus it might be possible to speculate that the SAR of 2.0 W/kg might be not sufficient to induce statistically significant changes in the cell proteome whereas the SAR of ≥5.0 W/kg might be sufficient to do so. Additionally, in the previous studies with 900 MHz GSM radiation, proteins spots were detected using silver staining whereas in the present study the DIGE-technique was applied. DIGE-technique is commonly considered to be more reliable and to produce less technical variability. In comparison with silver staining techniques, the use of DIGE-technique reduces the number of the observed false positive results.

Results of the present study are in agreement with Gerner et al. [9] who did not observe statistically significant changes in protein expression levels in proteomes of cells exposed to 1800 MHz GSM signal. Interestingly, they have detected changes in the rate of protein synthesis following long-term (8 hours) but not short term (1 hour) exposures. Our present study was not designed to determine effect on *de novo *protein synthesis observed by Gerner et al. [9].

In conclusion, our results suggest that, as expected, the proteomes of the same cell type (endothelium) but derived from different vascular beds (umbilical vein and brain microvasculature) express very different proteomes. However, the 1800 MHz GSM mobile phone radiation appeared to have no statistically significant effect on the proteome of HUVEC and HBMEC, when cells were exposed for 1 h at an average SAR of 2.0 W/kg and examined immediately after that.

## List of Abbreviations

2DE: two-dimensional electrophoresis; BVA: Biological Variation Analysis module in DeCyder; CHAPS: 3-[(3-Cholamidopropyl)dimethylammonio]-1-propanesulfonate; ddH_2_O: Double distilled water; DIGE: Difference Gel Electrophoresis; DTT: Dithioreitol; EA.hy926: Human endothelial cell line; EDA: Extended Data Analysis module in DeCyder; FDR: False discovery rate; GSM: Global System for Mobile Communications; HBMEC: Human Brain Microvascular Endothelial Cell; HUVEC: Human Umbilical Vein Endothelial Cell; IAA: Iodoacetamide; IEF: Isoelectric focusing; IPG: Immobilized pH gradient; PAGE: Polyacrylamide gel electrophoresis; PBS: Phosphate buffered saline; pI: Isoelectric point; PMSF: phenylmethylsulphonyl fluoride; SAR: Specific absorption rate; SDS: Sodium dodecyl sulfate; Tris-HCl: Tris(hydroxymethyl)aminomethane hydrochloride, Versene Chelating agent containing EDTA

## Competing interests

The authors declare that they have no competing interests.

## Authors' contributions

DL and RN designed the study; RN carried out the experiments; NK has provided radiation exposure equipment and radiation dosimetry, DL and RN have written and re-written the manuscript. All authors read and approved the final version of the manuscript.
